# Therapeutic Targets of KRAS in Colorectal Cancer

**DOI:** 10.3390/cancers13246233

**Published:** 2021-12-11

**Authors:** Shafia Rahman, Shimon Garrel, Michael Gerber, Radhashree Maitra, Sanjay Goel

**Affiliations:** 1 Department of Medical Oncology, Montefiore Medical Center/Albert Einstein College of Medicine, 1695 Eastchester Road Bronx, New York, NY 10461, USA; shafrahman@montefiore.org (S.R.); RMAITRA@montefiore.org (R.M.); 2 Department of Biology, Lander College For Men, 75-31 150th Street, Flushing, New York, NY 11367, USA; sgarrel@student.touro.edu; 3 Department of Biology, Yeshiva University, 500 West 185th Street, New York, NY 10033, USA; mgerber@mail.yu.edu

**Keywords:** colorectal cancer, *KRAS* mutation, targeted therapy

## Abstract

**Simple Summary:**

Colorectal cancer is among the most common cancers in the United States. The advancement in treatment and early diagnosis have enabled a reduction in mortality from the disease among the patients with early and localized disease; however, the survival continues to be dismal in the metastatic colorectal cancers. Understanding the biological and genetic factors is crucial is making the therapeutic strategy and improving survival outcomes. One of such critical steps is the understanding of the mechanism and development of therapeutic targets against metastatic colorectal cancers bearing the *KRAS* mutation.

**Abstract:**

Patients with metastatic colorectal cancer have a 5-year overall survival of less than 10%. Approximately 45% of patients with metastatic colorectal cancer harbor *KRAS* mutations. These mutations not only carry a predictive role for the absence of response to anti-EGFR therapy, but also have a negative prognostic impact on the overall survival. There is a growing unmet need for a personalized therapy approach for patients with *KRAS*-mutant colorectal cancer. In this article, we focus on the therapeutic strategies targeting *KRAS*- mutant CRC, while reviewing and elaborating on the discovery and physiology of *KRAS.*

## 1. Introduction

Colorectal cancer (CRC) is one of the most common cancers, with an estimated 1.5 million new cases and 52,980 deaths reported in 2021 in the United States, of which approximately 104,270 arise from the colon and the remainder from the rectum [1]. Although the mortality related to the CRC has been progressively declining since 1990, it continues to be the third most common cause of cancer death in both men and women, respectively, in the United States [2].

CRC arises through a multistep process involving accumulation of various epigenetic and genetic alterations [3]. The pathogenic mechanisms implicated in 80–85% of all CRC cases include microsatellite instability (MSI), CpG island methylator phenotype (CIMP), and chromosomal instability (CIN). The CIN is the most common pathogenic mechanism involved in the development of CRC. It results in the gain or loss of entire or large portions of chromosome resulting in karyotype changes within the cells. These karyotypic changes coupled with the mutations in the tumor suppressor and oncogenes (*APC*, *KRAS*, *DCC/SMAD4*, and *TP53)* activate oncogenic pathways critical to the pathogenesis of CRC [4]. Mutations in any of the four mismatch repair genes (*hMLH1*, *hMSH2*, *hMSH6*, and *hPMS2*) result in the microsatellite instability and leads to the development of the hereditary nonpolyposis colorectal cancer (HNPCC), also known as Lynch syndrome [5]. This genetic disorder is inherited as an autosomal dominant pattern and increases the risk of development of several cancers involving the colon, stomach, prostate, and small intestine [4]. The CpG island methylator phenotype is a unique subgroup of CRC. It is characterized by the epigenetic instability resulting in the hypermethylation of CpG island sites at the promoter regions that sequentially leads to transcriptional inactivation of tumor suppressor genes in CRC [5]. In the majority of the cases, one pathway is the predominant pathogenic pathway; however complex interplay in certain cases could be seen [6]. Apart from the above described pathogenic mechanisms, recent studies have shown the alteration in the various metabolic pathways including glycolysis, pyruvate oxidation, lactate oxidation, mitochondrial activity, and glutamine and cholesterol metabolism are involved in the initiation and progression of CRC [7].

The *RAS* gene family is mutated in approximately 30% of human cancers, with the *KRAS* isoform mutations being the major contributor [8,9,10]. In Colon cancers, approximately 45% of the cases carry a *KRAS* mutation [11,12]. These mutations in CRC are associated with aggressive tumor biology and poor survival. Moreover, the *KRAS* mutations in CRC lead to resistance to epidermal growth factor receptor (EGFR) directed therapies [13].

## 2. Discovery of RAS

Over six decades ago, Jennifer Harvey and Werner Kristen identified the two RAS gene, *HRAS* and *KRAS,* from the Harvey sarcoma virus and Kirsten sarcoma virus, respectively. The viral inoculum from leukemic rats was observed to induce sarcoma in newborn rats [14,15]. Later, Stehelin et al. proved that these proto-oncogenes could transform into oncogenes after acquiring mutations and these oncogenes can be virally transmitted [16]. Subsequently, in 1982, the human *HRAS* and *KRAS* oncogene sequences were ascertained in human bladder and lung cancer cell lines, respectively [17]. Later in 1983, the human sequences homologous to *NRAS* were described in human sarcoma cell lines [18]. Overall, the *RAS* family constitutes 36 human genes, but *KRAS, HRAS,* and *NRAS* by far are the most prominent ones involved in human cancer [19]. These high occurrences make *RAS* one of the most critical targets in oncology for drug development. 

## 3. Physiology of RAS

**Figure 1:**
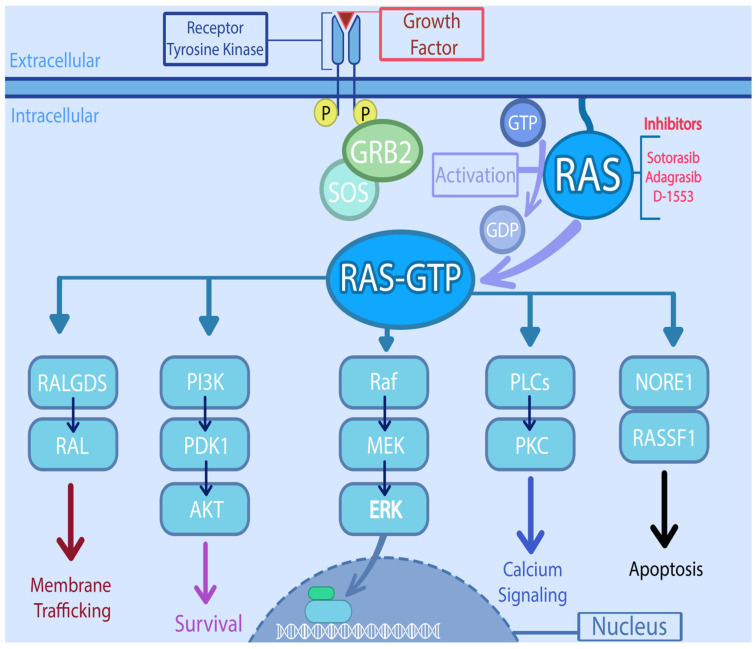
Signaling pathways downstream of RAS and potential targets. RAS directly activates the mitogen activated protein kinase (MAPK) cascade, through phosphorylation of Raf (Rapidly accelerated fibro sarcoma) which in turn phosphorylates MEK (Mitogen activated protein kinase kinase), which then phosphorylates MAPK. On the other hand, it also interacts with the PI3K (Phosphatidylinositol-4,5-Bisphosphate 3-Kinase)/AKT (serine/threonine protein kinase) pathway, either by PI3K interaction or through RAC1 which in turn activates p21-activated kinase (PAK), an AKT interacting protein. RAS also activates the RAL (RAS like proto-oncogene) which is involved in various steps of membrane trafficking. The PLCs (Phospholipase C) along with RINI/ABL plays important role in cytoskeletal remodeling. The activation of NORE1/RASSF1 is involved in cell cycle arrest and apoptosis.

*KRAS* is located at 12p12.1 and encodes a 188–amino acid residues [20]. The *RAS* family of genes acts as a universal confluence in the signal transduction of multiple intracellular pathways. *KRAS* and other RAS oncogenes are intracellular guanine nucleotide-binding proteins (G proteins) that belong to the family of small GTPases and function as GDP/GTP-regulated molecular switches [21]. It is activated by varying signals ranging from growth factors (epidermal growth factor receptor, platelet-derived growth factor receptor, insulin like growth factor, etc.), hormones, and cytokines to neurotransmitters [20]. Once activated, RAS moves from an inactivated, GDP-bound form, to activated GTP- bound state. The activation is catalyzed by guanine nucleotide exchange-factors (GEFs) and the conversion back to inactivated form by GTP hydrolysis mediated by GTPase-activating proteins (GAPs) [22]. The RAS activates multiple downstream pathways including the RAS-RAF-MEK-ERK pathway, which regulates cell cycle and proliferation [22]. Another pathway involved is PI3K-AKT-mTOR, which also promotes cell growth and suppresses apoptosis. The RAS-related protein (RAL) pathway and the tumor invasion and metastasis-inducing protein 1 (TIAM1-RAC1) are involved in intracellular vesicle trafficking, cytoskeletal organization, and tumor growth [23]. Thus, RAS proteins are essential regulators of the various aspects of normal cell growth and physiology and play a role in malignant transformation (Figure 1). Apart from playing critical role in the signal transduction involving multiple intracellular pathways, oncogenic *KRAS* is known to dysregulate various metabolic processes including glutaminolysis, glycolysis, and redox hemostasis promoting tumorigenesis and chemoresistance [24].

## 4. Mutations Involving RAS in CRC

RAS mutations have been associated with aberrant cell signaling that leads to tumor-promoting inflammation and play a key role in carcinogenesis by inducing an array of inflammatory cytokines, chemokines and accentuates tumorigenesis and invasiveness. The RAS mutations are common in CRC (~45%), with *KRAS* being the most prevalent (85%), followed by *NRAS* (15%) and *HRAS* (1%) [25]. The majority of the *KRAS* mutations in the CRC are located in codons 12 and 13 of exon 2 (80% are G12A, G12C, G12D, G12S, G12V, G13C, G13D), and less frequently in codon 61 of exon 3 (5% are Q61H, Q61L, and Q61R) and codon 146 of exon 4 (2% are A146T and A146V) [26]. Mutations in any of these codons promote the accelerated exchange of nucleotides, and a decrease in the binding of GAP. Either of these leads to increase GTP binding and *KRAS* activation. *KRAS* mutations also carry a predictive role for the absence of response to anti-EGFR therapy in metastatic CRC and thus have a negative prognostic impact as well [27,28]. 

## 5. Targeting *KRAS*

The therapeutic strategies under investigation to target *KRAS* mutations in CRC includes therapy directed towards mutant *KRAS*, targeting *KRAS*-membrane association, and the combined inhibition of downstream pathways. 

### 5.1. KRAS Directed

Several studies are being performed to identify molecules able to bind the mutated sites of *KRAS* or inhibit the synthesis at the DNA level of the mutated protein and subsequently blocking the activity of *KRAS*. 

### 5.2. AMG 510

AMG 510 (Sotorasib) is the first FDA-approved specific, irreversible inhibitor of *KRAS* G12C. It traps the *KRAS* in the inactive GDP-bound state [29]. AMG 510 has shown in the preclinical studies to inhibit phosphorylation of extracellular signal-regulated kinase (ERK), a critical downstream effector of *KRAS*, producing a durable complete tumor regression in mice bearing *KRAS* p.G12C tumors [30]. Based on the significant objective response rate and duration of response in a phase 1 trial CodeBreak100 (NCT03600883) [31], it was approved in locally advanced or metastatic NSCLC. Although the *KRAS* G12C is noted only in 1–3% of CRC, the recent promising clinical data breaks the assumption of *KRAS* being undruggable [32]. 

### 5.3. MRTX849

Another direct target of *KRAS* is MRTX849 (adagrasib). It works by irreversibly and selectively binding to *KRAS* G12C in its inactive state, blocking its signaling to other cells, thus preventing tumor cell growth and proliferation, leading to cancer cell death [33]. KRYSTAL-1 phase I/II clinical trial showed clinical activity in non-small cell lung cancer (NSCLC), CRC and other solid tumors such as pancreatic, endometrial, and ovarian cancers [34]. The FDA granted breakthrough therapy designation to MRTX849 for the treatment of patients with *KRAS* G12C-mutated non-small cell lung cancer patients that has previously received systemic therapy.

### 5.4. MRTX1133

MRTX1133. is another *KRAS* directed investigational drug. The preclinical studies have demonstrated that it selectively inhibits the *KRAS* G12D mutant forms, binds both the active and inactive forms and significant dose dependent tumor regression was noted in the animal models. The phase 1/1b study and phase 2 monotherapy trial in patients with NSCLC showed ORR of 45%, with mean duration of treatment being greater than 6 months [35,36].

Few other direct *KRAS* inhibitors targeting *KRAS* G12C mutation in phase 1 clinical trials include GDC-6036 (NCT04449874), JNJ-74699157 (NCT04006301), and D-1553 (NCT04585035) [37].

### 5.5. PLK-1 Inhibition

Polo-like kinase 1 (PLK1) is a serine/threonine kinase which plays a key role both in cell cycle progression via mitosis and DNA damage repair [38]. It has been found to be overexpressed in multiple cancer types including CRC [39]. Several studies have suggested correlation between Plk1 overexpression and poor prognosis. This has resulted in the development and emergence of PLK-1inhibtors as next generation anti-cancer therapy [40]. It has been observed that the RAS mutant cells are dependent on gene/proteins such as PLK-1, which are involved cell proliferation [41]. One such drug is Onvansertib, which is a selective competitive inhibitor PLK-1 inhibitor [42]. It is under clinical investigation as a second line therapy in metastatic CRC harboring *KRAS* mutation along with combination of FOLFIRI and bevacizumab (Clinicaltrials.gov Identifier: NCT03829410). Preliminary data from this Phase1b/2 study, which was presented at the American Society of Clinical Oncology (ASCO) Gastrointestinal symposium, showed that 42% of patients achieved a partial response (PR) and a durable response in 67% ranging from 6.1 months to 13.7 months [43].

### 5.6. KR12

Another promising drug for colorectal patients with either the G12D or G12V mutation is KR12. This agent is a pyrrole-imidazole polyamide indole-seco-CBI conjugate) which recognizes and alkylates the adenine residues on the template strand at codon 12 (GTT and GAT), exon 2 of mutated *KRAS*, producing strand cleavage which in turn decreases the proliferation rate of the CRC cell harboring G12D/G12 V mutation [44]. The growth suppression in G12D/G12V mutated CRC cells, ultimately induced senescence, and apoptosis. This effect was demonstrated in the preclinical study performed by Nagese et al. [44]. KR12 also induced significant tumor growth suppression in xenograft models, with low host toxicity in *KRAS*-mutated but not wild-type tumors, thus representing a promising agent against RAS mutated CRC with encouraging pre-clinical data. 

### 5.7. SHP2 Inhibition

Src homolofy-2 Domain containing protein tyrosine phosphatase-2 (SHP2) is encoded by human *PTPN11* gene and acts as a protein tyrosine phosphatase [45]. It is involved in various intracellular oncogenic signaling pathways, including the RAS/Raf/MAPK [46,47], PI3K/AKT [48], Jak/STAT [49], PD-1/PD-L1 [50], and mTOR pathways [51]. It functions as a protein tyrosine phosphatase that removes the tyrosine phosphorylation which is a crucial catalytic action and plays an important role in the multiple cellular functions including cellular proliferation, differentiation, and migration [52]. Overall, the primary oncogenic role of the SHP2 in the activation of the RAS/Raf pathway is to cause dephosphorylation of the tyrosine residues in the scaffolding proteins that result in the increased conversion of inactive RAS (RAS-GDP) to the activated RAS (RAS-GTP) [53]. This catalytic function also makes SHP2 a critical facilitator in acquired resistance of the RAS signaling reactivation, to overcome pharmacological inhibition [54]. These properties promote SHP2 inhibition as an attractive way to combat adaptive resistance, both as a monotherapy as well as and in combination with other agents such as, MEK inhibitors. Currently, four SHP2 inhibitors under investigation and are undergoing phase I clinical trials: JAB-0368 (NCT03518554), TN0155 (NCT03114319), RMC-4630 (NCT03634982), and RLY-1971 (NCT04252339). Table 1 depicts the *KRAS* directed drugs under clinical investigation involving metastatic CRC. 

## 6. Targeting Membrane Association

### Targeting of G4 Structures

**Figure 2:**
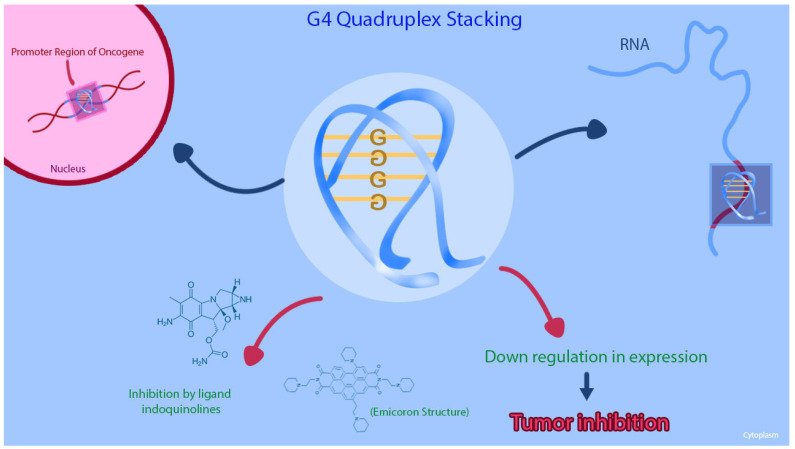
This figure shows that the G-quadruplex (G4) structures and the inhibition of the G4 structure on *KRAS* promoter region can cause downregulation of gene expression in CRC cell lines.

G-quadruplex (G4) structures are DNA tetraplexes that typically form in guanine-rich regions of genomes. Four guanine bases bind with Hoogsteen hydrogen bonds to form a guanine tetrad plane (G-quartet), and then two or more G-quartet planes stack on top of each other to form a G4 structure [55] Figure 2. The G4 structures are abundant in the promoter regions of many genes, including the regulation transcription of oncogenes and tumor suppressor genes [56,57,58]. Other than being reported on the *KRAS* human promoter DNA, G4 structures are found in RNA sequences, including the 5′ untranslated region (UTRs) of *KRAS* mRNA. Purro et al. identified natural alkaloids Indoloquinolines as potential G4-ligand compounds for targeting of *KRAS* in CRC [59,60]. They also synthesized a new molecule, namely EMICORON, which binds to the G4 structures on *KRAS*. Treatment with EMICORON, downregulated *KRAS* mRNA and protein expression in CRC cell lines, with decreased tumor volume in *KRAS*-mutated patient-derived xenografts [59]. It was further shown the EMICORON co- administration with FOLFIRI, improved the efficacy of chemotherapy in CRC-bearing mice [59].

## 7. Indirect Approaches

### 7.1. PDEδ Inhibition

Prenyl-binding protein PDEδ is crucial in maintaining the spatial organization of RAS during the activation of signaling pathway [61,62]. This creates a novel mechanism of indirectly targeting RAS signaling through inhibition of PDEδ. Zimmermann et al., developed Deltarasin, a small molecule inhibitor of PDEδ and demonstrated in the pre-clinical study that PDEδ inhibition by Deltarasin not only blocked the oncogenic RAS signaling but suppressed both in vitro and in vivo proliferation of human pancreatic ductal adenocarcinoma cells with highly prevalent oncogenic *KRAS* mutant genes [58]. Based on this pre-clinical data various small molecule inhibitors of PDEδ were generated. These inhibitors, such as Deltazinone 1 and Deltasonamide 1 and 2, competitively interact with the farnesyl-binding pocket of RAS [63,64]. Another pre-clinical study supporting the above strategy was done by Klein et al., [65]. They successfully demonstrated the inhibitory effect of PDEδ blockage on the proliferation and survival of *KRAS* mutant CRC cell lines. 

### 7.2. Targeting NRF2/Oxidative and Glutaminolysis

One of the major cellular changes which drive the proliferation in the cancer cells is the ability to induce metabolic reprogramming [63]. The normal cells induce glycolysis as a source of ATP in response to hypoxia. However, the cancer cells express exorbitant aerobic glycolysis promoting rapid cellular proliferation. This phenomenon is known as the Warburg’s effect [64]. One of the major sources of substrates for anerobic glycolysis is glutamine. Glutamine is an important amino acid necessary for the synthesizing glutamate via glutaminolysis, which in turn contributes to the tricarboxylic acid (TCA) cycle in the absence of glucose [65], thus making the cancer cells dependent on the glutamine-mediated TCA cycle for their energy needs. However, the rapid cellular proliferation and metabolism rate generates oxidative radicals deleterious for cell survival. The nuclear factor-erythroid 2 -related factor 2 (NRF2) and Kelch-like ECH-associated protein 1 (KEAP1) pathway protects the cells against oxidative and electrophilic stress and is tightly regulated under normal physiological conditions [66]. In cancer cells, apart from the enhanced glutaminolysis, dysregulation of NRF2/oxidative, inhibition of repressor genes, pathway promotes constant detoxification and transcription of cytoprotective proteins. Multiple experimental studies have shown that mutant *KRAS* enhances the activation of NRF2 antioxidant system and gene expression of the enzymes involved in the glutaminolysis, promoting tumorigenesis [67,68]. Given this critical role of glutaminolysis and NRF2/oxidative pathway, it has become an exciting therapeutic target to combat *KRAS* driven cancers. Furthermore, the glutaminase and NRF2 inhibitors have shown to enhance sensitivity of cancer cells to chemotherapy. Mukhopadhyay et al. showed that the NRF2 contributed to chemo-resistance in *KRAS* mutated pancreatic cancer cells and targeting these cell lines with glutaminase inhibitors sensitized the *KRAS* mutant cells to chemotherapy [69]. The glutaminase inhibitors under investigation so far include DON, JHU-083, BPTES, CB-839, and compound 968; however, the exuberant metabolic heterogenicity enhances the complexity of these targeted small molecule inhibitors [70,71]. Direct NRF2 inhibitor is another approach to sensitize *KRAS* mutant tumors to chemotherapy. One of the agents under investigation is brusatol and has shown promising data in pre-clinical studies [72,73].

### 7.3. Oncolytic Virus Induced Autophagy

Pelareorep is oncolytic reoviruses and has been under investigation as a therapeutic cancer-directed agent for over a decade [66]. Pelareorep can selectively infect the *KRAS* mutated CRC cells inducing lysis and promoting autophagy [67]. This was demonstrated in the pre-clinical study conducted by Maitra et al. [68]. In a phase I clinical trial, pelareorep was combined with FOLFIRI/Bevacizumab, which showed the combination was able to induce 50% partial response in the enrolled patient cohort [69]. Given the encouraging results, further exploration of this combination is needed as a modality for *KRAS* directed therapy.

## 8. Combined Inhibition of Downstream Pathways

The RAS/RAF/MEK/ERK and PI3K/AKT/mTOR are two of the most frequently dysregulated pathways in the human cancer biology [70]. These two pathways interact closely, not only sharing common inputs but are also activated by oncogenic RAS. These pathways also provide compensatory signaling when either one is inhibited [71]. This provides the scientific basis for combining therapeutic agents that could simultaneously block both the pathways and inhibit the downstream pathways involved in RAS signaling.

### 8.1. AZD4785

AZD4785 is a genetically engineered molecule which functions as an antisense oligonucleotide complementary to mRNA sequences of *KRAS* and selectively exhausts the intracellular *KRAS* mRNA and protein. The depletion of *KRAS* protein results in the repression of the subsequent signaling pathways and thus suppresses the cell proliferation. The *KRAS* depletion was not associated with any reciprocal feedback activation of MAPK pathway [72]. The other feature of AZD4785 is that its codon binding site on the *KRAS* mRNA is different from the mutation codon sites, thus it could be an effective strategy to target both wild and mutant type *KRAS*. Another pre-clinical study using AZD4785 on a panel of various tumor cell lines including CRC showed potent downregulation of mutant *KRAS* [73]. However, a Phase 1, dose escalation study of AZD4785 in patients with advanced solid tumors did not demonstrate sufficient *KRAS* lowering in target engagement, prompting the termination of therapeutic development (ClinicalTrials.gov Identifier: NCT03101839).

### 8.2. AZD6244

AZD6244 (Selumetinib) is an oral selective mitogen-activated protein kinase kinase (MAPKK, or MEK) pathway inhibitor and targets MEK1 and MEK2 [74]. The selumetinib interacts with MEK1/2 by turning MEK1/2 into their inactive conformational states. The inactive MEK 1/2 can undergo ATP and substrate binding, but disrupts the interactions required for extracellular signal-related kinase (ERK) activation. Pre-clinical studies with AZD6244 have shown regression of tumors both in *KRAS* wild-type BxPC3 pancreatic tumor xenograft model, as well as in colorectal, pancreatic, non-small cell lung, hepatocellular, and melanoma human xenograft models [75]. Bennouna et al. performed a Phase II study wherein AZD6244 therapy was compared to capecitabine monotherapy in CRC patients refractory to oxaliplatin and irinotecan. This study showed that selumetinib was well tolerated, and efficacy was comparable to capecitabine [76]. Apart from CRC, selumetinib has been tested in Phase II setting in various gastrointestinal tumor types including HCC, biliary cancer, and pancreatic cancer [77]. Currently, Phase II trials in combination with chemotherapy are undergoing in *KRAS* mutant CRC tumor types.

### 8.3. MEK and P13K/mTOR Combination

Combined Inhibition of downstream RAS signaling pathways such as MEK, P13K, and mTOR represents an effective strategy in *KRAS* mutant CRC. A phase 1 clinical study conducted by Shimizu et al. showed combined blockage using a PI3K pathway inhibitor in combination with a MAPK pathway inhibitor in advanced solid tumors including CRC observed tumor regression ranging between 2% and 64% [78]. A pre-clinical study supporting the above results was done by Pitt et al. where they demonstrated suppression of tumor progression in CRC cell lines and tumor xenografts models with combinatory PI3K/mTOR inhibitor PF-502 and the MEK1/2 inhibitor PD-901 [79]. Similar to these studies, there are numerous pre-clinical and early phase studies showing the synergistic effect of MEK and P13K/mTOR [80,81].

## 9. Developing Therapies

### 9.1. MiRNA as Potential Drug Candidates

MicroRNAs (miRNA) are small, approximately 20-nucleotide long, single stranded, non-coding RNA molecules and regulate gene expression by binding to complementary 3′ untranslated region (UTR) of a target gene leading to either degradation of mRNA or inhibition of translation [82]. Multiple studies have elucidated the critical role of miRNAs in cell proliferation, migration, invasion, apoptosis, and angiogenesis [83]. These miRNAs can function as either a tumor suppressor or an oncogene in the regulation pathway, depending on the cell context. For example, an miR-96 is upregulated in lung, prostate, bladder, colorectal, and breast cancer however the same miR-96 is downregulated in pancreatic cancer [84]. Chan et al. experimentally showed that miR-143 downregulation was associated with upregulation of *KRAS* protein in CRC cell lines. Upon treating CRC cell lines with miR-143, suppression of *KRAS* protein translation was observed. However, when miR-143 inhibitor was used it stimulated cell proliferation and increased the *KRAS* protein level [85]. In another study, Zhou et al. identified FAK and LAMB3 as targets of miRNA-1298 and observed that an miRNA-1298 mimic was noxious to CRC and NSCLC mutant cells in both vitro and in vivo conditions [86]. The regulatory role of miRNA on *KRAS* in various tumor types including CRC makes miRNAs a fascinating emerging drug therapy.

### 9.2. MYC Inhibition

The *RAS* and the *MYC* oncogenes interplay and interdependency play an essential role in driving cancer development [87]. Several studies in mouse models have demonstrated the importance of *MYC* in *KRAS*-driven oncogenesis and genetic suppression of *MYC* impairing the growth of *KRAS*-driven cancer cells [87,88]. The *MYC* oncoprotein in *KRAS* mutant cells is stabilized via increased *MYC* transcription and decreased protein degradation mediated by CDK 9 directed phosphorylation of *MYC* [89]. Thus, targeting the *MYC* oncogene could be a potential therapeutic strategy for *MYC*-dependent cancers such as *KRAS*-mutant CRC [90]. Voruciclib is a cyclin-dependent kinase (CDK) inhibitor and selectively inhibits cyclin-dependent kinase 4 (CDK4) and 6 (CDK6) [91]. This, in turn, blocks the phosphorylation of the retinoblastoma protein in early G1 phase, preventing the CDK-mediated G1-S phase transition leading to cell cycle arrest. The suppression of replication of DNA, in turn, inhibits tumor cell proliferation. The anti-neoplastic potential of voruciclib arises from its activity to inhibit the Cyclin-dependent Kinases. Wiley et al. presented the pre-clinical study at AACR virtual meeting [92]. The preclinical study showed that when cancer cell lines with *KRAS* mutations were treated with voruciclib, all cell lines had decrease in viability, and reduced *MYC* levels were noted. The ability of voruciclib to inhibit tumor growth in vivo was also tested in murine xenograft models injected separately with *KRAS* mutant CRC, NSCLC primary human cancer cells, wherein significant tumor growth inhibition (>50%) was observed at all doses of voruciclib [92]. This promising data from single agent voruciclib supports the hypothesis that the CDK9 inhibitor might synergize with the *KRAS* C12C inhibitors sotorasib and adagrasib.

### 9.3. T Cell-Mediated Therapy

Adoptive cell therapy (ACT) uses ex-vivo expanded tumor-reactive T-cells administered to an adequately prepared recipient [93]. This may be the future of RAS directed therapy [94]. Tan et al. in 2016 reported the first CD8+ T-cell response against mutant *KRAS* G12D in tumor-infiltrating lymphocytes (TILs) obtained from a patient with metastatic colorectal cancer. They reported objective regression of all seven lung metastatic lesions from underlying CRC after the infusion of *KRAS* G12D directed TILs [95]. RAS mutations represent ideal targets for immune-based treatments. The T cell mediated therapy represents unique modality that overcomes the many limitations of existing small molecule inhibitors, non-specific immune-based therapies, and passive vaccination trials. T cell-mediated therapy could represent the fascinating approach to overcome the prototypical “undruggable” RAS oncogene family.

## 10. Conclusions

In this review, we presented the promising advances in the development of *KRAS* directed therapy. *KRAS* directed therapy seems the most exciting approach, especially with the approval of the *KRAS 12C* directed agent in NSCLC. In the coming years, we are hopeful to have a similar agent in CRC based on the data from the preclinical and early phase trials. However, it is essential to consider that development of acquired resistance is inevitable. Apart from the *KRAS* directed therapy, combining it with a downstream pathway inhibitor such as CDK, immunotherapy, or chemotherapy needs further exploration. Ultimately, the tumor stratification is necessary for the success of the *KRAS* directed therapies. With our evolving knowledge regarding the heterogeneity of the *KRAS* mutated cancers and multiple subtypes of *KRAS* mutant forms, the precise selection of the patients for cancer-directed therapy will be necessary to ensure efficacy. Nonetheless, the future of *KRAS* directed therapy is promising. The data stimulate increasingly more effort to seek a better understanding of overcoming the long-time un-druggable target in oncology.

## Figures and Tables

**Table 1:** Ongoing *KRAS* directed clinical trials involving metastatic CRC.

Clinical Trial	Drug	Target	Cancer Type	Estimated Enrollment (N)	NCT ID
Phase I	MRTX849	*KRAS* G12C inhibitor	*KRAS* G12C mutant cancers	565	NCT03785249
Phase 1	*KRAS* TCR	Anti-*KRAS* G12D engineered T-cells	*KRAS* G12D Mutated cancer	70	NCT03745326
Phase 1	*KRAS* TCR	Anti-*KRAS* G12 V engineered T-cells	*KRAS* G12V Mutated cancer	110	NCT03190941
Phase 1	GDC-6036+/− Atezolizumab, Cetuximab, Bevacizumab, Erlotinib	*KRAS* G12C Mutation	Advanced or Metastatic Solid Tumors With a *KRAS* G12C Mutation	342	NCT0444987
Phase 1	BBP-398	SHP2 inhibitor	*MAPK* pathway or *RTK* driven advanced solid tumors	60	NCT04528836
Phase 1b/2	Onvansertib (PCM-075) + FOLFIRI + bevacizumab	PLK-1 inhibitor	Metastatic CRC with *KRAS* mutation	44	NCT03829410
Phase 1b/2	SX-682 +/−nivolumab	*CXCR1/2* inhibitor	Metastatic CRC, RAS mutated	53	NCT04599140
Phase 1	JNJ-74699157	*KRAS* G12 C	*KRAS* mutated advanced solid tumor	10	NCT0400630
Phase 1	mRNA-5671/V941 +/−pembrolizumab	*KRAS* vaccine	*KRAS* mutant CRC, NSCLC and PDAC	100	NCT03948763
Phase 1	D-1553	*KRAS* G12C inhibitor	*KRAS* mutated CRC and NSCLC	200	NCT04585035
